# Supplementary material for: Prescribing Patterns and Variations of Antibiotic Use for Children in Ambulatory Care: A Nationwide Study
Source: Antibiotics (Basel). 2022 Jan 31;11(2):189. doi: 10.3390/antibiotics11020189 (PMC8868088; doi:10.3390/antibiotics11020189)
Supplement: Supplementary file 1 [file antibiotics-11-00189-s001.zip › antibiotics-1544847-supplementary.pdf]

Supplementary Materials

Table S1. Top ten antibacterial agents among paediatric age groups in Hungary, 2017

Age groups	ATC code	Active substances	Prescriptions/ 100 children/ year	Percentage (%)	Cumulative percentage (%)
0-19 years	J01CR02	Amoxicillin and clavulanic acid	33.36	30.81	30.81
	J01FA10	Azithromycin	15.23	14.07	44.88
	J01DC02	Cefuroxime	11.90	10.99	55.87
	J01CA04	Amoxicillin	9.61	8.88	64.75
	J01DC10	Cefprozil	8.24	7.61	72.36
	J01DD08	Cefixime	6.36	5.87	78.23
	J01FA09	Clarithromycin	5.51	5.09	83.32
	J01CE02	Phenoxymethylpenicillin	4.34	4.01	87.32
	J01EE01	Sulfamethoxazole and trimethoprim	4.05	3.74	91.07
	J01DC04	Cefaclor	3.18	2.93	94.00
		Total	108.28	100.00	
0-4 years	J01CR02	Amoxicillin and clavulanic acid	59.83	32.54	32.54
	J01FA10	Azithromycin	25.21	13.71	46.25
	J01CA04	Amoxicillin	17.76	9.66	55.90
	J01DD08	Cefixime	17.08	9.29	65.19
	J01DC10	Cefprozil	14.34	7.80	72.99
	J01DC02	Cefuroxime	12.46	6.77	79.76
	J01DC04	Cefaclor	9.74	5.30	85.06
	J01FA09	Clarithromycin	8.09	4.40	89.46
	J01CE02	Phenoxymethylpenicillin	6.73	3.66	93.12
	J01EE01	Sulfamethoxazole and trimethoprim	6.40	3.48	96.60
		Total	183.90	100.00	
5-9 years	J01CR02	Amoxicillin and clavulanic acid	36.68	34.01	34.01
	J01FA10	Azithromycin	14.14	13.11	47.12
	J01DC02	Cefuroxime	13.53	12.55	59.67
	J01DC10	Cefprozil	9.73	9.02	68.69
	J01CA04	Amoxicillin	8.39	7.78	76.46
	J01CE02	Phenoxymethylpenicillin	5.74	5.32	81.78
	J01DD08	Cefixime	5.63	5.22	87.01
	J01FA09	Clarithromycin	5.17	4.79	91.80
	J01EE01	Sulfamethoxazole and trimethoprim	4.08	3.78	95.58
	J01DC04	Cefaclor	2.41	2.24	97.82
		Total	107.85	100.00	
10-14 years	J01CR02	Amoxicillin and clavulanic acid	18.08	25.78	25.78
	J01DC02	Cefuroxime	12.07	17.21	42.99
	J01FA10	Azithromycin	10.54	15.03	58.02
	J01CA04	Amoxicillin	7.38	10.51	68.53
	J01DC10	Cefprozil	5.44	7.76	76.29
	J01FA09	Clarithromycin	4.70	6.70	83.00
	J01CE02	Phenoxymethylpenicillin	3.09	4.41	87.40
	J01EE01	Sulfamethoxazole and trimethoprim	3.05	4.35	91.75
	J01DD08	Cefixime	1.63	2.32	94.07
	J01FF01	Clindamycin	1.11	1.58	95.65
		Total	70.15	100.00	
14-19 years	J01CR02	Amoxicillin and clavulanic acid	20.44	27.08	27.08
	J01FA10	Azithromycin	11.56	15.32	42.40
	J01DC02	Cefuroxime	9.65	12.78	55.18
	J01CA04	Amoxicillin	5.37	7.12	62.30
	J01FA09	Clarithromycin	4.23	5.60	67.90
	J01DC10	Cefprozil	3.85	5.11	73.01
	J01FF01	Clindamycin	3.21	4.26	77.27
	J01MA02	Ciprofloxacin	2.87	3.80	81.07
	J01EE01	Sulfamethoxazole and trimethoprim	2.82	3.73	84.80
	J01MA12	Levofloxacin	2.55	3.37	88.17
		Total	75.49	100.00	
